# Microclimate‐driven trends in spring‐emergence phenology in a temperate reptile (*Vipera berus*): Evidence for a potential “climate trap”?

**DOI:** 10.1002/ece3.8623

**Published:** 2022-02-10

**Authors:** Rebecca K. Turner, Ilya M. D. Maclean

**Affiliations:** ^1^ Environment and Sustainability Institute University of Exeter Penryn UK; ^2^ Present address: NERC UK Centre for Ecology & Hydrology Wallingford Oxfordshire UK; ^3^ Present address: Durrell Institute of Conservation and Ecology School of Anthropology and Conservation University of Kent Canterbury UK

**Keywords:** climate change, ecological trap, microclimate, reptile, species occurrence data, spring phenology

## Abstract

Climate change can not only increase the exposure of organisms to higher temperatures but can also drive phenological shifts that alter their susceptibility to conditions at the onset of breeding cycles. Organisms rely on climatic cues to time annual life cycle events, but the extent to which climate change has altered cue reliability remains unclear. Here, we examined the risk of a “climate trap”—a climatically driven desynchronization of the cues that determine life cycle events and fitness later in the season in a temperate reptile, the European adder (*Vipera berus)*. During the winter, adders hibernate underground, buffered against subzero temperatures, and re‐emerge in the spring to reproduce. We derived annual spring‐emergence trends between 1983 and 2017 from historical observations in Cornwall, UK, and related these trends to the microclimatic conditions that adders experienced. Using a mechanistic microclimate model, we computed below‐ and near‐ground temperatures to derive accumulated degree‐hour and absolute temperature thresholds that predicted annual spring‐emergence timing. Trends in annual‐emergence timing and subsequent exposure to ground frost were then quantified. We found that adders have advanced their phenology toward earlier emergence. Earlier emergence was associated with increased exposure to ground frost and, contradicting the expected effects of macroclimate warming, increased post‐emergence exposure to ground frost at some locations. The susceptibility of adders to this “climate trap” was related to the rate at which frost risk diminishes relative to advancement in phenology, which depends on the seasonality of climate. We emphasize the need to consider exposure to changing microclimatic conditions when forecasting biological impacts of climate change.

## Graphical abstract

In this study, we use novel microclimatic modeling techniques to assess whether advancement in the spring‐emergence phenology of the European adder has resulted in an increased exposure to frost, despite an overall reduction in frost risk under macrowarming. This study is among the first to demonstrate the existence of a climate trap, conceptually analogous to an ecological trap, and challenges traditional assumptions that macroclimatic warming will benefit species that require warmer conditions.

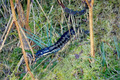

## 1 INTRODUCTION

Anthropogenic greenhouse gas emissions have already resulted in rapid macroclimate warming, with temperatures predicted to increase further by 1–5.7°C by 2100 (IPCC, 2021). Conventionally, climate change is expected to increase exposure to higher temperatures or reduce exposure to lower temperatures (Diele‐Viegas et al., 2020). However, species are also known to undergo phenological shifts, which may offset these changes in exposure; that is, by shifting the timing of biological events, species may be able to adapt to accommodate climate change (Forrest, 2016). Nevertheless, in rapidly changing environments, the cues that animals use to regulate behavior can decouple from longer‐term fitness and reproductive outcomes (Robertson & Chalfoun, 2016; Schlaepfer et al., 2002). This phenomenon is proposed to give rise to an “ecological trap,” whereby organisms become constrained by their evolutionary responses to cues at a cost to their fitness (Gilroy & Sutherland, 2007). It is thus possible that phenological shifts may also become maladaptive under climate change by increasing exposure to suboptimal conditions later in the season. In these scenarios, species may be experiencing what we refer to as a “climate trap.”

While there are high‐profile examples of climate change leading to biotic asynchrony (e.g., Both et al., 2006; Visser et al., 1998), the effect of phenological advancement on an organism’s climatic exposure and associated long‐term fitness is largely unknown. Assessing the potential for climate change to alter fitness, and the particular mechanisms that underpin this, is therefore a necessary precursor to fully understanding the vulnerability of species to climate change. However, a major challenge to determining the prevalence of climate traps has been in quantifying the climatic conditions as organisms experience them. Until recently, most studies have relied on coarse‐scale spatial and temporal resolution climatic data derived from weather stations (Araújo et al., 2006; Kearney & Porter, 2009, 2017). However, such data can substantially differ from the climatic conditions in the microenvironments in which organisms reside, and may thus provide a poor proxy for conditions experienced in nature (Bramer et al., 2018; Lembrechts et al., 2019). Approaches that consider the magnitude of microclimatic exposure are thus crucial for making reliable predictions of species survival and population persistence under climate change (Beaumont et al., 2005; Kearney & Porter, 2009; Taylor et al., 2020).

In recent years, efficient and accurate approaches to modeling microclimatic conditions have emerged. The R package “NicheMapR” incudes a suite of tools for mechanistic modeling of heat and mass exchange between organisms and their environments (Kearney & Porter, 2017). The tool enables predictions of hourly above‐ and belowground conditions from meteorological, terrain, vegetation, and soil data, though requires model pre‐adjustments of important “mesoclimate” effects (elevation‐associated lapse rates, wind sheltering, coastal influences, and cold air drainage) and estimates of terrain variables (slope, aspect, and hill shade). Maclean et al. (2017) developed a series of functions for such mesoclimate and terrain adjustments. This extended the model of Bennie et al. (2008), released as an R package “microclima” (Maclean et al., 2019), which includes functionality to account for canopy shading effects. Combined, the two models provide a unified framework for modeling the microclimatic conditions that organisms experience (Kearney et al., 2020). Here, we make use of these advances to quantify the risk of a climate trap.

**FIGURE 1:**
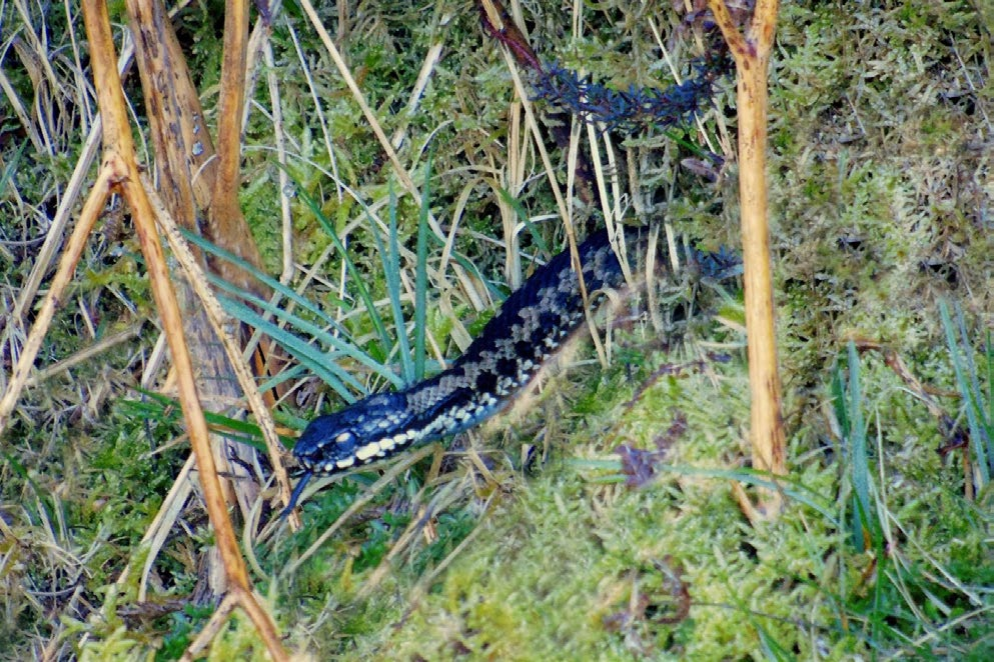
European adder (*Vipera berus*) is one of many temperate‐zone poikilotherms to exhibit seasonal patterns of behavior, such as emergence from hibernation in the spring

Poikilotherms such as amphibians and reptiles are excellent study subjects when assessing climate‐driven phenological changes (Blaustein et al., 2001; Carey & Alexander, 2003; Henle et al., 2008; Taylor et al., 2020). Our study focuses on a temperate reptile, the European adder (*Vipera berus*; Figure 1). Temperate‐zone species exhibit seasonal patterns of behavior (Bauwens & Claus, 2019) that are strongly related to their (micro)climatic environment (Angilletta et al., 2002; Obbard & Brooks, 1987), as determined by inter‐annual variability in seasonal temperatures (Rugiero et al., 2013). The functionality of amphibian and reptile immune systems can also fluctuate seasonally, with lower functionality during hibernation and at emergence (Kobolkuti et al., 2012). Consequently, they are sensitive to critical climate events during these periods, such as sudden and prolonged periods of frost (Bauwens, 1981; Costanzo & Lee, 2013; Layne et al., 1998; Storey, 2006; Voituron et al., 2002). Indeed, it is not uncommon for late‐season frosts to occur in temperate regions, particularly after mild winters (Benard, 2015). For adders that emerge early from hibernation, prolonged exposure to frost is suboptimal. It can result in direct mortality (Andersson & Johansson, 2001) or may constrain adders’ ability to sustain the higher energy demands needed to revive physiological functions after hibernation (see Brischoux et al., 2016). Early emergence may thus increase the risk of getting caught in a “climate trap.” Evidence for maladaptive phenological responses to climate change, consistent with a climate trap effect, has been implicated in herpetological research previously. For example, temporal shifts in breeding (Beebee, 1995; Blaustein et al., 2001; Forchhammer et al., 1998; Gibbs & Breisch, 2001) and hibernation cycles (Blouin‐Demers et al., 2000; Gardner et al., 2019; Rugiero et al., 2013) have been documented, both of which can reduce fitness and reproductive success (Abney et al., 2019; Benard, 2015; Combes et al., 2018; Donnelly & Crump, 1998; Luiselli et al., 2018; Sheridan et al., 2017). Shifts in annual spring‐emergence could therefore have major implications for adders due to a heightened risk of reduced fitness and survival during the early spring‐emergence period (Bauwens & Claus, 2019). The timing of full emergence is thus a critical decision for adders, with potential for adders to fall into a climate trap.

**FIGURE 2:**
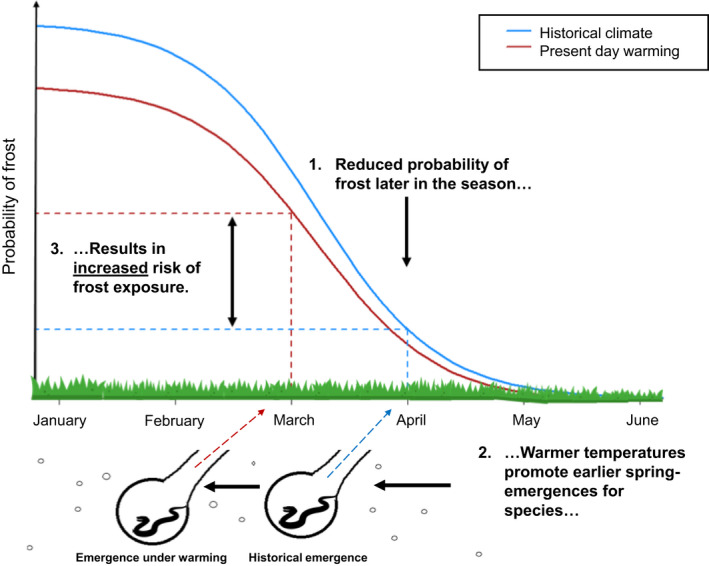
Conceptual diagram of a "climate trap.” Under climate change, warmer temperatures reduce the overall likelihood of spring frost, but the probability of frost remains greatest earlier in the season. Ambient warming may also result in phenological advancement, such as earlier emergence from hibernation in temperate‐zone poikilotherms that rely on temperature cues to time spring‐emergence. If the advancement in phenology overcompensates for the reduction in unfavorably cold temperatures, a species will fall into a “climate trap,” experiencing increased exposure to colder, thermally suboptimal environments despite ambient warming

Here, we quantify spatial and temporal variation in the risk of climate traps for adders across Cornwall, UK, as follows. First, we collated historic records of spring adder sightings and used microclimate models to calculate belowground hibernacula temperatures at each sighting location. We then explored relationships between early sightings and various absolute and accumulated temperature‐related thresholds to derive temperature metrics that most accurately predicted emergence. To examine the effects of climate change on adder spring‐emergence phenology, we estimated long‐term trends in the timing of emergence using temperature thresholds. Next, we used microclimate models to calculate the number of hours of exposure to ground frost and thus assess whether earlier emergence was associated with increased risk of encountering unfavorable conditions. Finally, we examined the spatial variation and long‐term trends in ground frost exposure to examine what drives the risk of being caught in a climate trap. If climate traps are arising, a tendency for warming temperatures to heighten the risk of exposure to ground frost should be detectable. Overall, we hypothesized that frost risk is greater earlier in the year and that warming temperatures across years not only reduce frost frequency overall but also trigger earlier emergence. The rate at which phenology advances relative to the rates of frost reduction determines the risk of getting caught in a climate trap (see Figure 2).

## 2 MATERIALS AND METHODS

### 2.1 Study system

The European adder is a small, venomous snake with an extensive geographic range spanning Europe and Asia (Bauwens & Claus, 2019). From October to March, adders hibernate in overwintering dens (“hibernacula”) approximately 25–100cm underground (Appleby, 1971; Viitanen, 1967). Adders are capable of surviving only very short exposures of subzero temperatures (Andersson & Johansson, 2001; Bulakhova et al., 2017) and select hibernacula that thermally buffer against the colder winter temperatures (Weatherhead, 1989), allowing individuals to maintain a low but relatively constant body temperature above their critical minimum (~5–7°C) (Appleby, 1971; Brischoux et al., 2016; Kobolkuti et al., 2012; Spellerberg, 1982; Viitanen, 1967). Aboveground activity typically commences from early March, where adders bask in direct sunlight to raise their body temperatures to within an optimal range for breeding (Appleby, 1971; Bauwens & Claus, 2019). Males tend to emerge earlier from hibernation than females, basking for long periods of time near their hibernacula to increase the likelihood of successful spermiogenesis (Prestt, 1971; Viitanen, 1967). Mating commences between April and May (Bauwens & Claus, 2019; Prestt, 1971).

This study was conducted in Cornwall, the most south‐westerly region of the UK mainland. The county is surrounded by the North Atlantic Ocean on three sides and has a highly oceanic climate—low inter‐annual variability in temperature, mild, wet winters, relatively cool summers, and prolonged and varied seasonal transitions. This makes the region an appropriate landscape to study the effects of climate‐driven phenological shifts.

### 2.2 Adder sighting data

Historical records of adder sightings in the form of occurrence data were obtained from three sources: (1) The Environmental Records Centre for Cornwall and the Isles of Scilly (ERCCIS, 2019), (2) the ERICA database (Cornish Biodiversity Network, 2019), and (3) the Record Pool (Amphibian and Reptile Groups UK & Amphibian and Reptile Conservation Trust, 2019). Dead sightings, duplicates, and records dated from June to September were removed, leaving 344 records. Records were observations of adders collected by a variety of recorders (incl. expert surveyors and the general public) and included observations from structured surveys, legacy datasets, and ad hoc sightings, though information on the sampling methods associated with each record was not discernible from the dataset. Data providers performed the initial screening of records using automated computer checks, and a species expert, typically the County Recorder for Cornwall, verified and validated records. We used only validated records. Records were available from 1983 to 2019, though there were no observations in 1986 and 1999. For all other years, available records ranged from 1 to 41 (*M* = 9.8, *SD* = 9.8) observations per year, with most observations (64%) occurring between 2010 and 2019.

### 2.3 Predicting emergence

We predicted spatiotemporal patterns of emergence as follows. First, for each spring adder location in each year, we computed the temperatures of adder hibernacula at hourly intervals using a microclimate model. Second, we assumed that emergence is related to an accumulation of degree‐hours. Third, we related adder observations in the spring to accumulated degree‐hours to compute the threshold at which emergence occurs, but because each observation occurs at some unknown time period after emergence, we computed the cumulated degree‐hours at the time for sighting separately, ranked each sighting by their cumulative degree‐hour value, and selected the 5th percentile value as the trigger of emergence. Lastly, to calculate the spatiotemporal trends in emergence, we ran the microclimate model separately for each adder sighting location in the dataset and year to calculate the date on which the cumulative degree‐hour threshold for emergence was reached. Additionally, we explored the sensitivity of our results to assumptions made about the climatic drivers of emergence and percentile values triggering emergence (see supporting information).

In the absence of radiative heat provided by the sun, body temperatures closely match surrounding air temperature (Campbell & Norman, 2012) and we assumed therefore that adders will attain the average temperatures of their hibernacula such that emergence in the spring is related to the average temperatures at a soil depth of 10 and 50 cm (i.e., the approximate depth of the upper regions of hibernacula used by adders in the UK in which sensitivity to temperature variation is greatest (Appleby, 1971)). Accordingly, hourly temperatures at depths of 10 and 50 cm were calculated using the R package “microclima” (Maclean et al., 2019), which integrates with the soil heat model of NicheMapR (Kearney & Porter, 2017). The package contains a series of functions for computing mesoclimatic variation and the effects of terrain and vegetation on microclimate at specified heights belowground (or aboveground). Mesoclimate effects are determined by fitting thin‐plate spline models to coarse‐resolution hourly differences between land and sea temperature data with elevation, coastal exposure upwind, and mean coastal exposure in all directions included as covariates. The thin‐plate models are then applied to derive land–sea temperature differentials for specific locations at fine resolution using higher‐resolution terrain data, in this case, at 100‐m resolution. Wind speed, directions, and sea‐surface temperature data were obtained from the National Weather Surface National Centres for Environmental Prediction (Kanamitsu et al., 2002) and air temperatures from the UK Met Office (Met Office, 2018). Digital terrain data, used to apply the thin‐plate spline models, are sourced by the package itself.

After adjusting for mesoclimatic effects, soil temperatures were computed by dividing the soil into nine vertical layers down to a depth of 200 cm. Substrate temperature profiles were then calculated using an Adams predictor–corrector method to solve the nine simultaneous first‐order ordinary differential equations of the heat budget for each soil layer. Depth‐specific soil moisture, which in turn affects the heat capacity of soil, was simulated by linearizing the differential equation for flow in space, and using a Newton–Raphson procedure to solve the non‐linear equations through time (see Campbell, 1985). Full details of the soil model used in this study, including empirical validation of the method, are provided in Kearney and Porter (2017) and Kearney et al. (2020). The net radiant energy supplied to the surface heat layer was computed by downscaling radiation data sourced from EUMETSAT Satellite Application Facility on Climate Monitoring (Posselt et al., 2014). Total incoming shortwave radiation was partitioned into its direct and diffuse components using the “microclima” function “difprop,” which implements the approach described by Skartveit et al. (1998). Outgoing radiation was computed from temperature and sky emissivity, itself determined from net incoming radiation, with values at night derived using spline interpolation. Radiation was downscaled by accounting for local terrain and canopy cover, the latter assumed to be that typical of short grass (see Kearney et al. (2020) for details). The models were tested against 164,748 temperature readings from 106 locations obtained between 2010 and 2014 using iButton thermochrons (mean error = 1.21°C; root mean square error = 1.63 °C; see Maclean et al. (2017), Maclean et al. (2019) for further details).

We assumed that emergence occurs once accumulated degree‐hours in the hibernacula are reached. In computing accumulated degree‐hours, a base temperature of 7°C was assumed as this represents the critical minimum temperature for adders, below which locomotion is hindered (Kobolkuti et al., 2012; Spellerberg, 1982; Taylor et al., 2020; Viitanen, 1967). The accumulated temperature method is most commonly used to predict the development of organisms and assumes a linear relationship between temperature and development rates. It has also been used to predict reptile and amphibian phenology (e.g., DeGregorio et al., 2017; Lovich et al., 2012; Obbard & Brooks, 1987; Woodley, 2013). However, because the reliability of this method is unknown, in addition to using a cumulative degree‐hour threshold model, we also investigated three other alternative cues for adder emergence. These were as follows: (1) a sharp rise in accumulated temperatures, where emergence would be preceded by a period in which accumulated degree‐hours increased most rapidly; (2) an assumption that emergence is instead related to a collapse in hibernacula temperature gradients, such that emergence occurs once temperatures at 10‐cm soil depth exceeded those at 50‐cm soil depth, thereby reversing the normal winter temperature–depth profile; and (3) an assumption that emergence is triggered by a rolling‐mean aboveground critical air temperature of 10°C degrees being reached. Further details of the methods used and justification for the choice of these cues are presented in the supporting information. However, it should be noted that in general, soil heat storage is significant, and thus itself determined by the accumulation of radiative heat occurring over several months (Campbell, 1985). Thus, while our four alternative cues represent significantly divergent mechanisms by which emergence may occur, in practice they are closely correlated. As such, we only present those for a threshold of accumulated temperature in the main text (see the supporting information for the results of the alternative cue scenarios).

For each adder sighting during spring (January 1 to May 31), within years with which suitable climate data were available (1983–2017), the cumulative degree‐hours prior to the sighting were computed. Since all sightings relate to individuals that had already emerged from hibernation, but emergence may have occurred at some unknown period prior to the sighting, we ranked each sighting by their cumulative degree‐hour and maximal soil temperature values and selected the 5^th^ percentile value as that which triggered emergence (see, e.g., Prodon et al., 2017). This emergence threshold was then used to estimate the annual‐emergence timing in each spring from 1983 to 2017 for each location in the final dataset. To account for the possibility that the timing of emergence is not well represented by using a 5th percentile value, we used sensitivity analyses to establish the extent to which our results were sensitive to alternative percentile value (see the supporting information for alternative results using 2.5th and 10th percentile thresholds). While the choice of value affected our estimates of emergence timing, the overall effects on spatiotemporal trends were qualitatively quite similar using this accumulated temperatures cue. For each live adder sighting location, an hourly time series of soil temperature at 10‐ and 50‐cm depths belowground was computed for each year from 1983 to 2017. We then computed the predicted date of emergence at each site in each year.

### 2.4 Frost risk after emergence

To estimate frost risk, we computed ground surface temperatures at hourly intervals at each sighting location in each year using the microclimate modeling procedures described above. Frost exposure was considered to occur if ground temperatures were ≤0°C after emergence. The relative exposure to ground frost was then quantified as the total number of hours after the emergence in which ground temperatures were ≤0°C between January and June. To assess inter‐annual variation in conditions and adder phenology, trends in the rates of predicted overall spring ground frost, emergence, and post‐emergence ground frost exposure were computed, respectively, using linear mixed models with year as a fixed factor. To account for variation between locations, we included each sighting location as a random factor.

### 2.5 Climate traps

To test for potential climate traps, we pooled all emergence and ground frost exposure data and used a Pearson product–moment correlation to assess the relationship between emergence timing and subsequent exposure to ground frost at each site. We also assessed trends in the risk of experiencing a climate trap across sites over the study period. To do so, site‐specific trends in emergence timing and exposure to ground frost after emergence were calculated for each location using linear models. The model coefficients were then plotted on a map to depict the magnitude and direction of long‐term trends in exposure to ground frost after emergence at each site. To illustrate the mechanism underpinning climate trap formation, plots for one inland and one coastal site with divergent post‐emergence ground frost exposure trends were generated for a typical cold (1987) and warm (1995) year. We defined coastal sites as those which situated within 3km of the coastline. To examine the differences in the types of sites where climate traps were arising, we performed a logistic regression, which regressed the number of post‐emergence ground frost hours against the site location (inland/coastal). The analyses described above were performed in R programming language version 4.1.2. (R Core Team, 2021) and QGIS version 3.6.1 Noosa (QGIS Development Team, 2021).

## 3 RESULTS

### 3.1 Trends in annual spring‐emergence and ground frost

**FIGURE 3:**
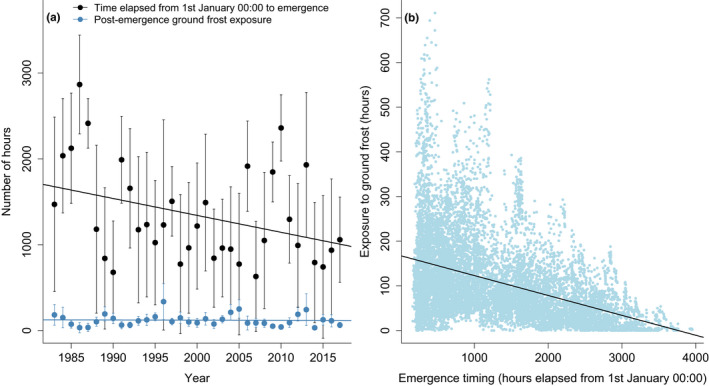
(a) Mean, standard deviation, and trends in annual *Vipera berus* emergence timing and post‐emergence exposure to ground frost in Cornwall, UK, from 1983 to 2017. (b) Relationship between *V. berus* emergence timing and post‐emergence exposure to ground frost across 344 sites in Cornwall between 1983 and 2017 (Pearson's *r* = −.44)

Rates of annual spring ground frost at adder sites in Cornwall reduced over the duration of the study, reducing from an average of 385 h of ground frost in 1983 to 146 h by 2017 (b ± *SE* = −5.6 ± 0.2, $\chi_{1,33}^{2}$ = 910, *p* < .001). Adder emergence was predicted to occur when accumulated degree‐hours above 7°C reached 21. Over the duration of the study, macroclimate warming appeared to have resulted in hibernacula warming. The timing of predicted emergence advanced by 28 (± 0.8) days ($\chi_{1,33}^{2}$ = 1211, *p* < .001; Figure 3a) over the study period, though there was divergence among sites. Over the study duration, there was a minor reduction in the average number of ground frost hours after emergence in Cornwall (b ± *SE* = −5.5 ± 2.7 h, $\chi_{1,33}^{2}$ = 4.2, *p* = .04; Figure 3a), but, again, variation between sites. We also found that earlier adder emergence was significantly associated with an increased post‐emergence exposure to ground frost (*r* = −.44, *p* < .001, *N* = 12,040; Figure 3b).

### 3.2 Climate traps

**FIGURE 4:**
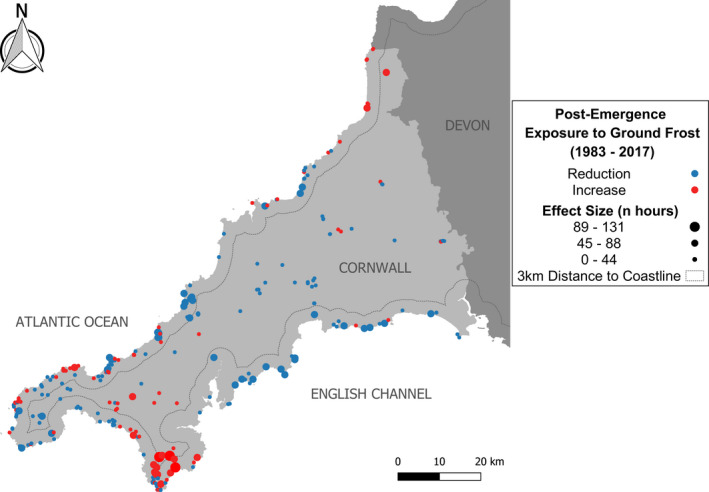
Trends in annual post‐emergence ground frost exposure for *Vipera berus* at sites in Cornwall, UK, with known historical occupancy (1983–2017) (*n* = 344)

**FIGURE 5:**
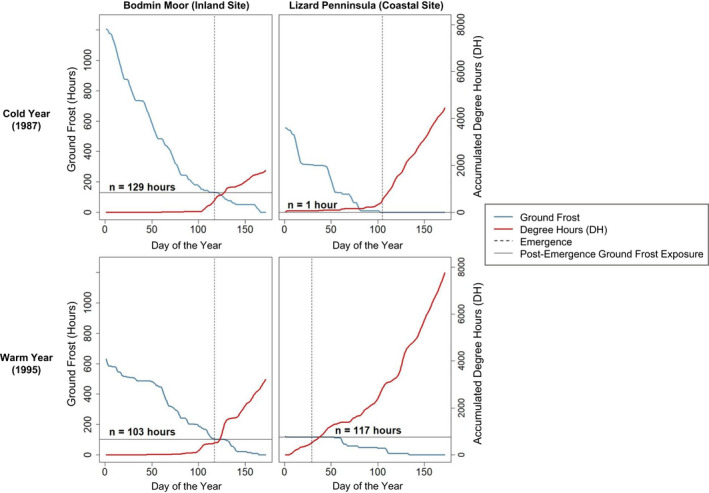
Estimated *Vipera berus* emergence timing, temperature accumulation (degree‐hours), and ground frost depletion in warm and cold years at two *V*. *berus* sites in Cornwall, UK, with known historical occupancy. The results for one site situated on Bodmin Moor (an inland site with reduced post‐emergence ground frost between 1983 and 2017) and one site situated on the Lizard Peninsula (a coastal site with increased post‐emergence ground frost between 1983 and 2017) are presented

Site‐specific trends in exposure to ground frost following emergence revealed divergence among sites. We identified 85 sites where adders were potentially at risk of experiencing a climate trap. At these sites, adder emergence appeared to have significantly advanced over the study period, and the amount of ground frost experienced by adders at these sites showed increasing trends. Possible climate trap sites appeared to be mostly coastal sites (70%) as rates of post‐emergence ground frost exposure were significantly reduced at inland sites (*b* ± *SE* = −24 ± 2 h, *F*_2,12038_ = 176, *p* < .001) compared with coastal sites. Coastal sites in general also showed greater variability in the magnitude of change in adder post‐emergence exposure to ground frost. While ground frost appears to have reduced at most sites (64%), there were a clear trend toward increased exposure at southern coastal sites, and a general trend toward reduced exposure at inland sites (Figure 4). Whether sites show a trend toward greater or lesser exposure to ground frost post‐emergence appeared to be dictated by the rate at which spring ground frost depletes over the course of the year, relative to the rate at which temperature (degree‐hours) accumulates (Figure 5). Moreover, the amount of variation in emergence timing between warm and cold years differed across sites. As shown in Figure 5, adder emergence timing during a typical warm year was predicted to result in far greater advancement at a coastal site than that at an inland site. At the coastal site, a pronounced mismatch between the accumulated temperatures experienced underground and the risk of encountering frost aboveground due to warming directly resulted in increased frost exposure at emergence.

## 4 DISCUSSION

Climate change‐related phenological shifts may threaten species persistence if the conditions for an ecological trap arise. Climate change has been implicated in dramatic population declines and referenced in ecological trap studies (Ahmadi et al., 2019; Araújo et al., 2006; Robertson et al., 2013; Van Dyck et al., 2015). However, research quantitatively examining these associations has been hampered by difficulty in accounting for the conditions directly experienced by organisms. To our knowledge, no previous studies have demonstrated long‐term trends in the microclimatic conditions experienced by organisms depending on the timing of emergence from hibernation across numerous locations. Here, we used contemporary microclimate modeling procedures to quantify the risk of a “climate trap.” We demonstrate a mechanism by which an ecological trap may arise under macrowarming in a temperate reptile, offering new insights into the extent to which climatic changes lead to ecological traps.

Adders in the UK have advanced their spring‐emergence phenology (Gardner et al., 2019). Our study extends earlier work as the warming of adder hibernacula under climate change appeared to have resulted in earlier spring‐emergence between 1983 and 2017 in Cornwall. Earlier emergence from hibernation has also been well documented in amphibians (Parmesan, 2007; While & Uller, 2014) and is the most common phenological response to climate change in reptiles (Prodon et al., 2017). For instance, long‐term studies have shown advancement in spring‐emergence by approximately 19 days for the Asp viper (*Vipera aspis)* over a 25‐year time period (Rugiero et al., 2013), which is comparable to the rate of advancement shown here. However, changes to cycles of activity for adders had not previously been associated with adverse consequences (Phelps, 2008).

To improve upon current understanding of species responses to climate change, we sought to determine whether advancements in annual spring‐emergence were likely to be a maladaptive response to warming for adders. Our results revealed that earlier emergence was associated with an increased exposure to ground frost for adders, despite an overall reduction in the amount of ground frost occurring over the study period. Given their thermally sensitive ecology (Herczeg et al., 2007; Herczeg, Saarikivi, et al., 2007; Spellerberg, 1982), increased exposure to ground frost is almost certainly a fitness cost to adders. While adders may be partially freeze‐tolerant, frequent exposure and reduced body condition at emergence reduce the likelihood of survival; thus, there is potential that prolonged periods of freezing temperatures in the spring could result in direct mortality (Andersson & Johansson, 2001; Bauwens & Claus, 2019; Brischoux et al., 2016). Taken together, this indicates that earlier emergence in the spring increases adders’ risk of encountering thermally unfavorable conditions. Accordingly, variation in emergence timing is likely to result in lower fitness outcomes. For adders, and other temperate poikilotherms, the timing of emergence from hibernation is the result of a trade‐off between risks (e.g., exposure to unfavorable conditions) and potential advantages (e.g., extension to the active period, higher reproductive success) (Blouin‐Demers et al., 2000). It is therefore not surprising that adders would tend toward earlier emergence if the climate cues driving emergence are normally indicative of higher fitness and reproductive success. While climate change‐related shifts in phenology can increase the aboveground activity time for poikilotherms (Green, 2017; Menzel et al., 2006; Parmesan & Yohe, 2003), unfavorable (cold) conditions for these species are more likely to occur earlier in the year (Augspurger, 2013; Inouye, 2008). Therefore, a decoupling of the above‐ and belowground temperatures in the spring under macrowarming could also increase exposure to unfavorable conditions (Kearney, 2020). Unless hibernating species recognize the increased risk of poorer conditions earlier in the season, some organisms may experience lower survival or reproductive outcomes, and therefore risk of getting caught in a climate trap.

The impact of climate change‐related shifts in spring activity may fluctuate between populations. The evidence presented here indicates that the risk of getting caught in a climate trap can also vary, as adder sites in Cornwall experienced divergent trends. Trends toward reduced ground frost exposure were observed at some sites, though at others this trend was completely reversed and directly contrasted the general reduction in ground frost occurring in the spring in Cornwall. In these instances, adders were at a higher risk of encountering ground frost after emergence and thus at risk of getting caught in a climate trap. Phenological advancements can sometimes offer advantages, but at other times have deleterious effects due to asynchronies between climate cues and selective conditions. Indeed, warmer spring temperatures have led to earlier breeding in other species (Abney et al., 2019; Combes et al., 2018; Sheridan et al., 2017) at a potential cost to fitness (e.g., altered body size [Ficetola & Maiorano, 2016; Sheridan et al., 2017]) and reproductive success (e.g., reduced fecundity, [Benard, 2015]). Maladaptive shifts in phenology, as indicated here, may therefore also threaten other amphibians and reptiles under climate change.

The ability to predict climate traps strongly depends on understanding the conditions in which they arise. In this study, adders in Cornwall experienced variable rates of surface temperature accumulation and ground frost depletion once they had emerged from hibernation. The relationship between these variables appeared to be an important determinant of the magnitude of exposure to unfavorable conditions. In general, coastal areas, particularly on the south coast of the study region, exhibited strong evidence of possible traps arising. The climate at these locations is susceptible to strong maritime influences, and here, we found that ground frost depleted at a slower rate than temperature accumulated during the spring. In general, the climate and seasonality of coastal areas in Cornwall are less predictable than inland as warm, prevailing south‐westerly winds, which results in relatively few frost events. Frost events typically occur when winds are northerly/north‐easterly and are as likely to occur in spring as in winter (Maclean et al., 2017). Moreover, rates of climate warming in coastal areas are generally slower than those of inland sites due to the influence of the sea, which has a higher heat capacity and slower thermal inertia than the land. In consequence, earlier emergence in coastal regions seemed particularly detrimental to adders as they experienced prolonged exposure to ground frost. This study has thus advanced current understanding of ecological traps by demonstrating a means of identifying the specific conditions that suggest its existence using novel microclimate modeling techniques.

For many smaller, ground‐dwelling species with poor dispersal capability, the characteristics of their microclimatic environments are likely to determine the cues that regulate behavior. The thermal requirements of poikilotherms, such as adders, are known physiological drivers of behavior and habitat choices. Species with innate mechanisms that regulate behavior will be the most vulnerable to becoming caught in an ecological trap, while those that make use of experience‐based learning are likely to be more robust against traps (Kokko & Sutherland, 2001). In this study, we did not account for the impact of phenotypic plasticity in a species ability to adapt in landscapes affected by rapid anthropogenic climate change. Plastic responses are likely to only be effective for mitigating against the effects of climate change in long‐lived species inhabiting highly variable environments (Schlaepfer et al., 2002), though this may present an insightful area of research.

Several theories exist regarding drivers of spring‐emergence in reptiles. These include mating systems (Gregory, 1974; Olsson & Madsen, 1996), body condition (Graves & Duvall, 1990), fixed schedules (Weatherhead, 1989), and hibernacula microclimate (Blouin‐Demers et al., 2000; Crawford, 1991). In this study, we tested two empirically supported theories for emergence as a response to (1) reaching a threshold of temperature acclimation and; (2) an increase in ambient temperatures (see supporting information). We acknowledge that the climate metric tested may actually be one of many cues used by adders to time emergence from hibernation. However, the precise cues, and their relative biological and temporal importance to adders, remain an open line of enquiry in research. Snake depth in the hibernacula and underground temperature gradients may also influence emergence timing (Blouin‐Demers et al., 2000; Carpenter, 1953; Lang, 1971). The “gradient collapse” theory (see Viitanen, 1967) has received some empirical support over the years (e.g., Blouin‐Demers et al., 2000; Macartney et al., 1989; Sexton & Hunt, 1980; Sexton & Marion, 1981), although in a highly maritime climate such as that of Cornwall, this is unlikely to be a reliable driver of emergence as daytime near‐surface ground temperatures, even in the middle of winter, can exceed those at greater depth (Kearney et al., 2020).

The present study has provided the first demonstration of a mechanism by which the adder could become constrained by environmental cues, potentially at a cost to their fitness, in landscapes undergoing rapid change. Adders appear to have undergone a gradual advancement in spring‐emergence toward earlier times in the season. Advances in phenology at some locations were sufficient to heighten the risk of exposure to ground frost following emergence, despite the overall prevalence of frosts diminishing in response to warming. These combinations of findings are indicative of a climate trap (Robertson & Hutto, 2006). The likelihood of adders falling into this climate trap appears to be related to the rate at which frost diminishes relative to phenological advancement. This risk was most pronounced at locations with typically less seasonality in frosts, particularly in strongly maritime‐influenced coastal regions. Exposure to adverse climatic conditions can only realistically be determined with the recently available microclimatic modeling tools. Together with the consideration of species physiology and ecology, microclimate modeling procedures could also help to improve the efficacy of conservation efforts (Griffis‐Kyle, 2016). For instance, identifying key attributes of topography in areas where climate is least likely to have an adverse impact may be helpful in informing landscape‐scale decisions (Donaldson et al., 2017; Isaak et al., 2017). Conversely, identifying where species are most at risk may be helpful in informing in situ approaches. Carefully timed habitat management, for example, can be used to alter microclimatic conditions and thus buffer populations of species against the adverse effects of climate change (Greenwood et al., 2016). By accounting for conditions as organisms experience them, we are likely to significantly enhance our understanding of ecological responses to climate change.

## CONFLICT OF INTEREST

The authors have no conflicts of interest to declare.

## AUTHOR CONTRIBUTION

**Rebecca K. Turner:** Conceptualization (equal); Data curation (equal); Formal analysis (equal); Methodology (equal); Writing – original draft (lead); Writing – review & editing (equal). **Ilya M. D. Maclean:** Conceptualization (equal); Data curation (equal); Formal analysis (equal); Methodology (equal); Writing – review & editing (equal).

## Supporting information

Supplementary Material

## Data Availability

Data are available from the Dryad digital repository: https://doi.org/10.5061/dryad.05qfttf4h.
