# Supplementary material for: Microclimate‐driven trends in spring‐emergence phenology in a temperate reptile (Vipera berus): Evidence for a potential “climate trap”?
Source: Ecol Evol. 2022 Feb 10;12(2):e8623. doi: 10.1002/ece3.8623 (PMC8831210; doi:10.1002/ece3.8623)
Supplement: Supplementary file 1 — Supplementary Material [file ECE3-12-e8623-s001.pdf]

Supporting Information

Microclimate-driven trends in spring-emergence phenology in a temperate reptile (*Vipera berus*): Evidence for a potential 'climate trap'?

Rebecca. K. Turner[1,2,3]*, Ilya. M. D. Maclean[3]

[1] Current address: NERC UK Centre for Ecology & Hydrology, Wallingford, Oxfordshire, UK.

[2] Current address: Durrell Institute of Conservation and Ecology, School of Anthropology and Conservation, University of Kent, Canterbury, UK.

[3] Primary affiliation: Environment and Sustainability Institute, University of Exeter, Penryn, TR10 9FE, UK.

Author Identification (in the order of the authors as they are listed above)

1.ORCiD iD https://orcid.org/0000-0001-5159-8266

2. ORCiD iD https://orcid.org/0000-0001-8030-9136

**Corresponding author*: rkt4@kent.ac.uk

Summary

This supporting information presents a sensitivity analysis in which alternative cues for *Vipera berus* (adder) emergence from hibernation are used to predict annual adder emergence timing and quantify post-emergence frost exposure at sites in Cornwall, United Kingdom, with known historical occupancy between 1983 and 2017, and thereby quantify the risk of a 'climate trap' (see the main text for Turner & Maclean, *Ecology and Evolution*).

1 Emergence Threshold Sensitivity Analysis

1.1 Methods

To test the robustness of our results to the assumptions made about the triggers of *Vipera berus* (adder) emergence, we investigated three other alternative climate cues derived from the literature as potential cues for adder spring-emergence in addition to the cue presented in the main text. In the main text, we assumed that emergence occurred once accumulated temperatures above a threshold (accumulated degree-hours above base 7°C) in the hibernacula was reached. Here we consider three alternative cues: (1) we assumed that emergence would follow a sharp rise in accumulated temperatures due to increasing spring temperatures (Macartney, et al., 1989; DeGregorio et al., 2017). Here, we assumed that emergence would be preceded by a period in which accumulated degree-hours increased most rapidly, as determined from the gradient of the accumulated degree-hours curve. (2) We assumed that emergence is instead related to a collapse in hibernacula temperature gradients (Viitanen, 1967; Litzgus, et al., 1999; Macartney et al.,1989). We therefore estimated the timing of adder emergence as the hour at which the spring 2.5-day moving-average of temperatures at 10 cm soil depth exceeded the 2.5-day moving-average of those at 50 cm soil depth, thereby reversing the normal winter temperature-depth profile. (3) We examined emergence as a response to an above-ground critical air temperature, consistent with observations of *Vipera spp*. peak emergence (Viitanen, 1967; Brito, 2003; Gardner et al., 2019). This is one of the most widely recorded environmental covariates in adder emergence surveys and has accurately predicted spring-emergence in other reptiles (e.g. Macartney et al., 1989; Rugeiro et al., 2013; DeGregorio et al., 2017). The most widely reported environmental variable associated with emergence timing in adders in the literature is maximum air temperature. Peak emergence timing has been correlated with maximum air temperatures between a range of 6°C to 13°C for *Vipera spp*., with temperatures between 8°C and 12°C the most frequently reported (Viitanen, 1967; Appleby, 1971; Brito, 2003; Gardner, et al., 2019). We therefore selected a median maximum critical temperature value of 10°C air temperature, whereby the timing of adder emergence was predicted at the hour at which the spring 2.5-day moving-average of maximum air temperature reached 10°C.

In the case where emergence is assumed to be related to accumulated temperatures above a critical threshold, the threshold was determined using the dates of adder observations in the sightings dataset. For each live adder sighting during spring (1st January to 31st May), the cumulative degree-hours prior to the sighting were computed. Since all sightings related to individuals that had already emerged from hibernation, but emergence may have occurred at some unknown period prior to the sighting, we ranked each sighting by their cumulative degree-hour. In the main text we selected the 5th percentile value as that which triggered emergence. Here we test the possibility that the timing of emergence is not well-represented by the 5th percentile value and compare our results with those obtained also using the 2.5th and 10th percentile values. Under these scenarios, adder emergence was predicted to occur when accumulated degree-hours above 7°C reached 9 and 241, respectively as opposed to 21 when the 5th percentile was used in the main text.

To test for potential climate traps under each of the alternative cue scenarios, we pooled all emergence and frost risk data and used a Pearson product-moment correlation

to assess the relationship between emergence timing and subsequent exposure to ground frost emergence at each site using the emergence and frost risk data derived from each cue separately. We also assessed trends in the risk of experiencing a climate trap across sites over the study period under each cue scenario. To do so, site-specific trends in emergence timing and exposure to ground frost after emergence were calculated for each location using linear models. The model coefficients were then plotted on maps to depict the magnitude and direction of long-term trends in exposure to ground frost after emergence at each site for each cue scenario. The number of sites demonstrating evidence of a climate trap (i.e. both an advancement in emergence timing and increased post-emergence ground-frost exposure) were derived between all cue scenarios. The number of potential climate traps at coastal and inland locations were compared. Coastal sites were defined as situating within 3km of the coastline, where as inland sites were located further than this distance from the coastline. To examine the differences in the types of sites where climate traps were arising, we performed a logistic regression which regressed the number of post-emergence ground frost hours against the site location (inland/coastal). To illustrate the mechanism underpinning climate trap formation using the accumulated degree-hours cues, plots for one inland and one coastal with divergent post-emergence frost exposure trends were generated for a typical cold (1987) and warm (1995) year. When using the gradient collapse cue and maximum critical air temperature cue, plots for a year with mild warming (2012) were also derived. In the main text, results are presented for the cumulative-degree-hours 5th percentile threshold scenario. Below we also present the results for the alternative (2.5^{th} and 10th percentile) threshold scenarios, as well as the results using other alternative cues.

1.2 Results

1.2.1 Trends in emergence timing and ground frost exposure

As reported in the main text, adder emergence timing was shown to have advanced in Cornwall across years under all alternative cues triggering emergence and under all alternative percentile values selected for defining the threshold triggering emergence (Table S1). The largest advancement (52 days) was predicted when 10°C critical air temperature critical temperature threshold was used. By contrast, an advance of just 12 days was predicted when a collapse in hibernacula temperature gradients was assumed to trigger of emergence.

There was notable variation between the estimates of exposure to ground frost trends depending on the assumed cue or percentile threshold used to predict adder emergence timing (Table S2). Most estimates were comparable to the results presented in the main text depicting a reduction in post-emergence exposure to ground frost for adders. The exception to this was the case where the 10th percentile was used as the threshold for accumulated temperature. In this scenario, no change in exposure over the study duration was predicted. By contrast, by using 2.5^{th} percentile accumulated degree-hours threshold, the greatest reduction in ground-frost exposure was predicted. Overall, when a 10°C critical air temperature was used to predict emergence, the risk of experiencing ground frost was predicted to increase over the study period (by 67 hours). This was the only scenario in which post-emergence ground frost exposure was predicted to increase. For comparison, the 5th percentile accumulated degree-hours threshold presented in the main text provided

a median estimate of post-emergence ground frost exposure for adders. Trends in emergence timing and post-emergence exposure to ground frost for all scenarios are shown in Figs S1-4. Irrespective of assumptions made about the trigger of or threshold for emergance, there was, as would be expected, a negative relationship between the timing of adder emergence and post-emergence ground frost exposure rates (Table S3). This relationship was typically strongest when a critical temperature threshold was used to predict emergence timing. The 5th percentile accumulated degree-hours threshold presented in the main text produced a median estimate of this relationship.

Table S1. Effects of assumptions made about the cues of *Vipera berus* spring emergence on the trends on *V. berus* emergence timing in Cornwall, United Kingdom, from 1983 – 2017. Model outputs from linear mixed-model analyses.

**Climate cue**	**Estimated change in emergence timing (days)**	**S.E. (days)**	**Chi-Square**	***Sign.***
**Cue 1**: Accumulated degree-hours above threshold (5th percentile)	-28	1	1211	$P < 0.001$**
Accumulated degree-hours above threshold (2.5th percentile)	-21	1	828.69	$P < 0.001$**
Accumulated degree-hours above threshold (10th percentile)	-28	0.5	3240	$P < 0.001$**
**Cue 2**: Sharpest rise in accumulated degree-hours	-27	1	1175	$P < 0.001$**
**Cue 3**: Below-ground temperature gradient collapse	-12	0.5	576	$P < 0.001$**
**Cue 4**: Critical air temperature	-52	1	1972	$P < 0.001$**

*** alpha < 0.001.*

Table S2. Effects of assumptions made about the cues of *Vipera berus* spring emergence on the trends on *V. berus* post-emergence exposure to ground frost in Cornwall, United Kingdom, from 1983 – 2017. Model outputs from linear mixed-model analyses.

**Climate cue**	**Estimated change in ground frost exposure (hours)**	**S.E. (hours)**	**Chi-Square**	***Sign.***
**Cue 1**: Accumulated degree-hours above threshold (5th percentile)	-6	2.7	4.2	$P = 0.04$*
Accumulated degree-hours above threshold (2.5th percentile)	-39	4	112	$P < 0.001$**
Accumulated degree-hours above threshold (10th percentile)	0	0	1.3	$P = 0.26$
**Cue 2**: Sharpest rise in accumulated degree-hours	-13	1	203	$P < 0.001$**
**Cue 3**: Below-ground temperature gradient collapse	-2	1	5	$P = 0.03$*
**Cue 5**: Critical air temperature	+67	3	481	$P < 0.001$**

** alpha < 0.05, ** alpha < 0.001.*

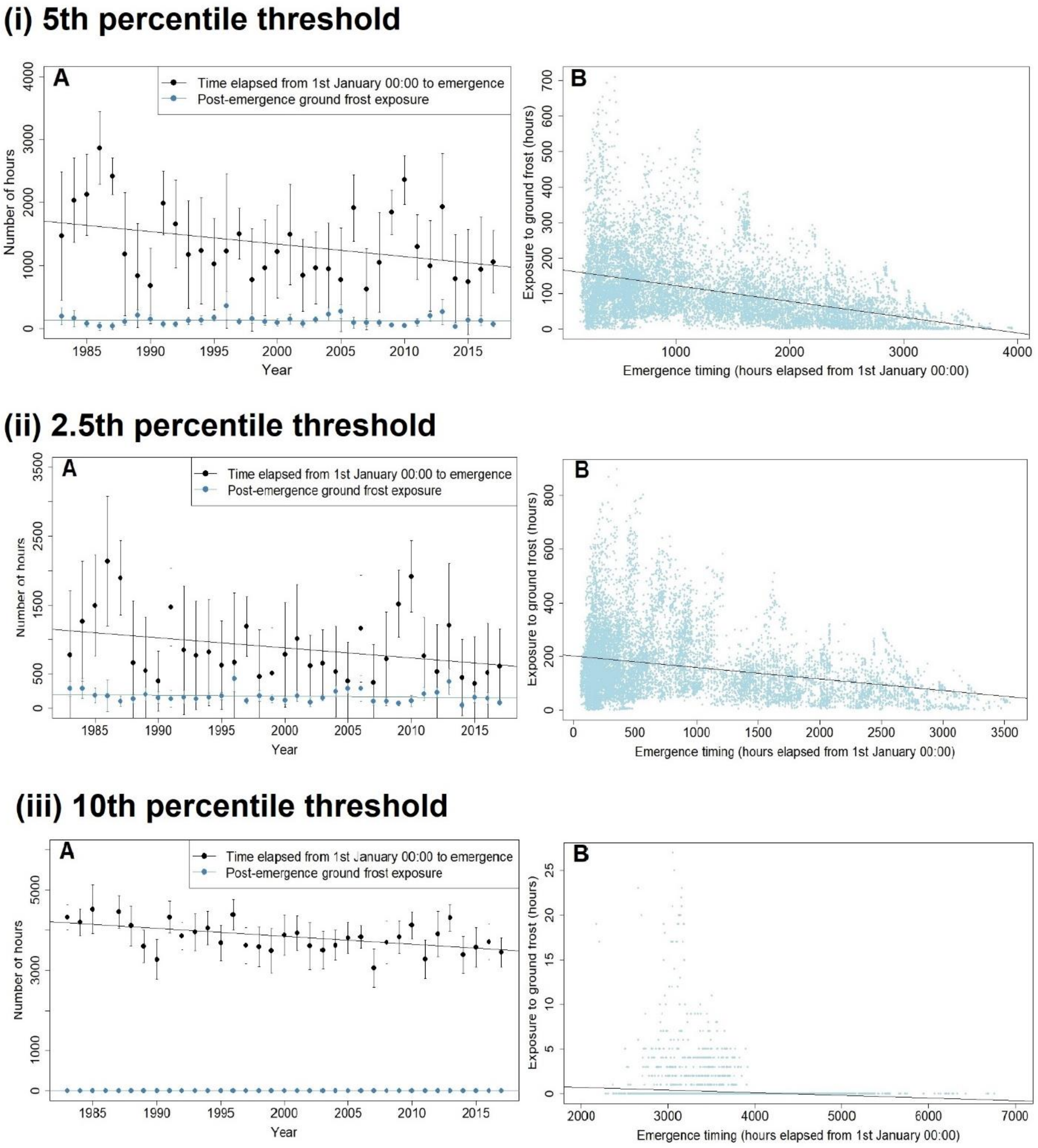

Figure S1. *Vipera berus* emergence timing and post-emergence exposure to ground frost using the (i) 5th percentile, (ii) 2.5th percentile, and (iii) 10th percentile threshold values of accumulated degree-hours as the cue for *V. berus* spring-emergence. (A) Mean, standard deviation and trends in annual *V. berus* emergence timing and post-emergence exposure to ground frost in Cornwall, United Kingdom, from 1983 to 2017. (B) Relationship between *V. berus* emergence timing and post-emergence exposure to ground frost across 344 sites in Cornwall between 1983 and 2017.

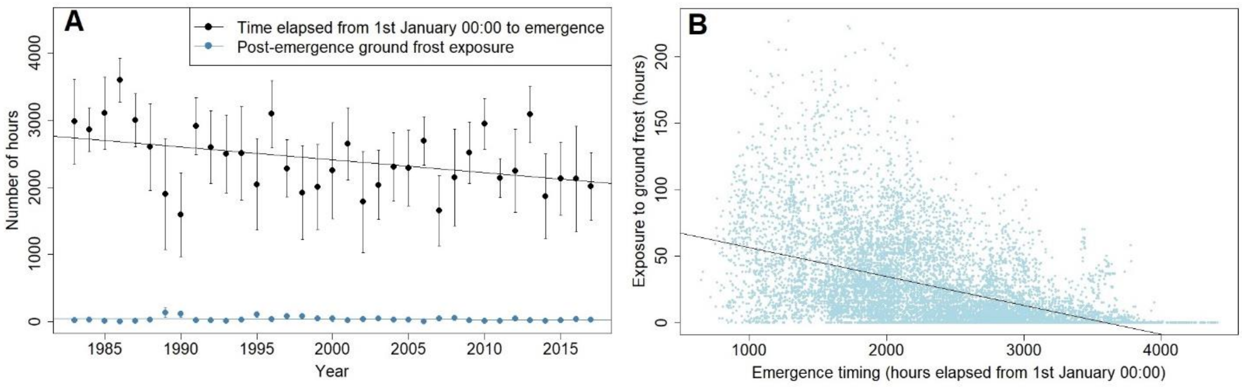

Figure S2. *Vipera berus* emergence timing and post-emergence exposure to ground frost using the sharpest rise in accumulated degree-hours as the cue for *V. berus* spring-emergence. (A) Mean, standard deviation and trends in annual *V. berus* emergence timing and post-emergence exposure to ground frost in Cornwall, United Kingdom, from 1983 to 2017. (B) Relationship between *V. berus* emergence timing and post-emergence exposure to ground frost across 344 sites in Cornwall between 1983 and 2017.

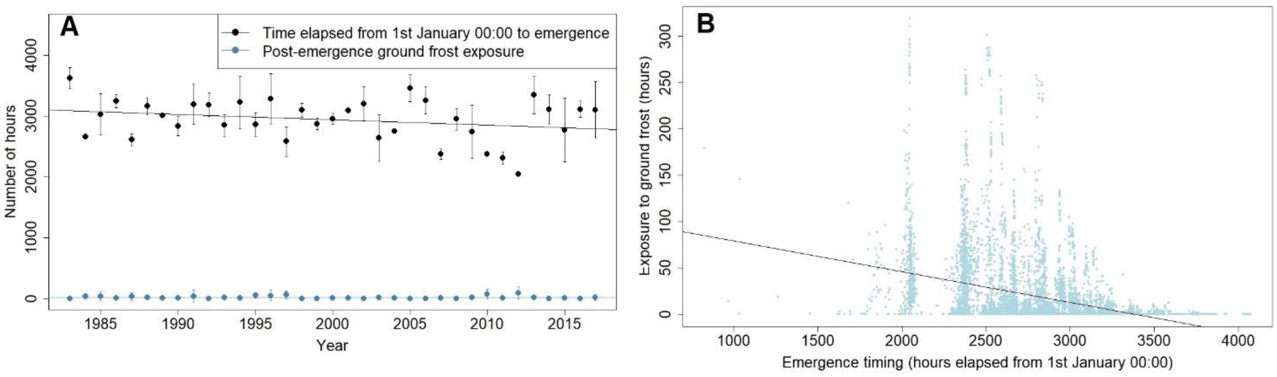

Figure S3. *Vipera berus* emergence timing and post-emergence exposure to ground frost using a below-ground temperature gradient collapse as the cue for *V. berus* spring-emergence. (A) Mean, standard deviation and trends in annual *V. berus* emergence timing and post-emergence exposure to ground frost in Cornwall, United Kingdom, from 1983 to 2017. (B) Relationship between *V. berus* emergence timing and post-emergence exposure to ground frost across 344 sites in Cornwall between 1983 and 2017.

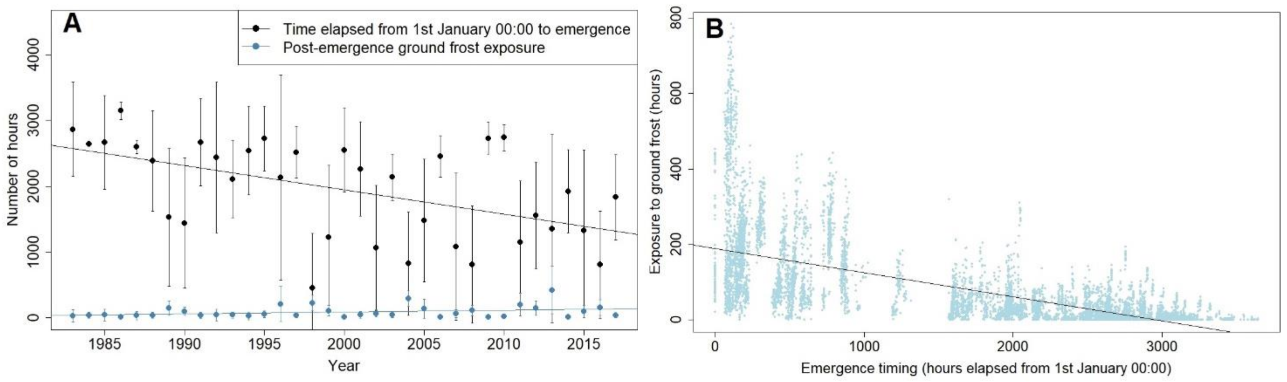

Figure S4. *Vipera berus* emergence timing and post-emergence exposure to ground frost using a 10°C critical air temperature as the cue for *V. berus* spring-emergence. (A) Mean, standard deviation and trends in annual *V. berus* emergence timing and post-emergence exposure to ground frost in Cornwall, United Kingdom, from 1983 to 2017. (B) Relationship between *V. berus* emergence timing and post-emergence exposure to ground frost across 344 sites in Cornwall between 1983 and 2017.

Table S3. Effects of assumptions made about the cues of *Vipera berus* spring emergence on Pearson's correlation between the timing of emergence and the number of hours of exposure to ground frost thereafter (N = 12040). All relationships were significantly (P < 0.001**) negative.

**Emergence trigger**	**R**
**Cue 1**: Accumulated degree-hours above threshold (5th percentile)	-0.44
Accumulated degree-hours above threshold (2.5th percentile)	-0.29
Accumulated degree-hours above threshold (10th percentile)	-0.14
**Cue 2**: Sharpest rise in accumulated degree-hours	-0.51
**Cue 3**: Below-ground temperature gradient collapse	-0.39
**Cue 4**: Critical air temperature	-0.68

1.2.2 Climate Traps

Irrespective of assumptions made about the trigger of emergence, the magnitude and predicted trends of post-emergence ground-frost exposure differed between sites. There were also notable differences depending on the assumed cue or percentile threshold used to predict adder emergence timing (Fig S5). Generally, coastal sites were less likely to exhibit a decrease in exposure than inland sites, and sites on the south coast were most likely to exhibit an increase. Adders at inland sites were predicted to experience significantly less ground frost following emergence using the steepest increase in degree-hours cue (b ± s.e. = -9 ± 0.6 hours, $F_{1,12038}$ = 228, P < 0.001), the 2.5th percentile threshold (b ± s.e. = -20 ± 2.4 hours, $F_{1,12038}$ = 65, P < 0.001), marginally using the 10th percentile threshold (b ± s.e. = -0.14 ± 0.02 hours, $F_{1,12038}$ = 36, P < 0.001), and the critical maximum air temperature cue (b ± s.e. = -15 ± 2 hours, $F_{1,12038}$ = 52, P < 0.001). However, rates of post-emergence ground frost were significantly higher at inland sites when the collapse in hibernacula

gradients was used to predict emergence (b ± s.e. = 22 ± 0.7 hours, $F_{1,12038}$ = 1083, P < 0.001). The magnitude of change in rates of ground frost exposure also appeared greatest for coastal sites (Fig S5). Although, the number of sites exhibiting an increase in exposure to ground frost did depend on the assumptions made about the trigger of emergence. The number of potential climate traps (i.e. sites demonstrating both an advancement in emergence timing and increased post-emergence ground-frost exposure) identified using the alternative cues to predict adder spring-emergence are presented in Table S4. The highest number of climate traps were identified using a critical air temperature threshold to predict emergence, resulting in 73% of sites showing potentially showing evidence of a climate trap formation. Conversely, the fewest number of climate traps were detected when a collapse in the hibernacula temperature gradient was used to predict emergence, with 31 potential climate traps identified using this cue. Under the majority of emergence cue scenarios, climate traps were more likely to occur at a coastal site than an inland site. However, this was not the case when using the 2.5th percentile value of accumulated degree-hours cue, which resulted in a higher proportion of inland sites identified as climate traps. The number of detectable climate trap sites were comparable between the different percentile thresholds used to predict adder emergence timing. In the main text, using the 5th percentile accumulated degree-hours threshold produced a median estimate of number of potential climate traps identified amongst all the cues and thresholds used to predict emergence.

Table S4. Effects of assumptions made about the cues of *Vipera berus* spring emergence on the number of detectable potential climate traps across all sites in Cornwall, United Kingdom, with known historical occupancy (n = 344).

**Emergence trigger**	**Number of potential climate trap sites**		
	Inland	***Coastal***	***N***
**Cue 1**: Accumulated degree-hours above threshold (5th percentile)	25	60	85
Accumulated degree-hours above threshold (2.5th percentile)	37	21	58
Accumulated degree-hours above threshold (10th percentile)	30	79	109
**Cue 2**: Sharpest rise in accumulated degree-hours	7	36	43
**Cue 3**: Below-ground temperature gradient collapse	8	23	31
**Cue 4**: Critical air temperature	61	189	250

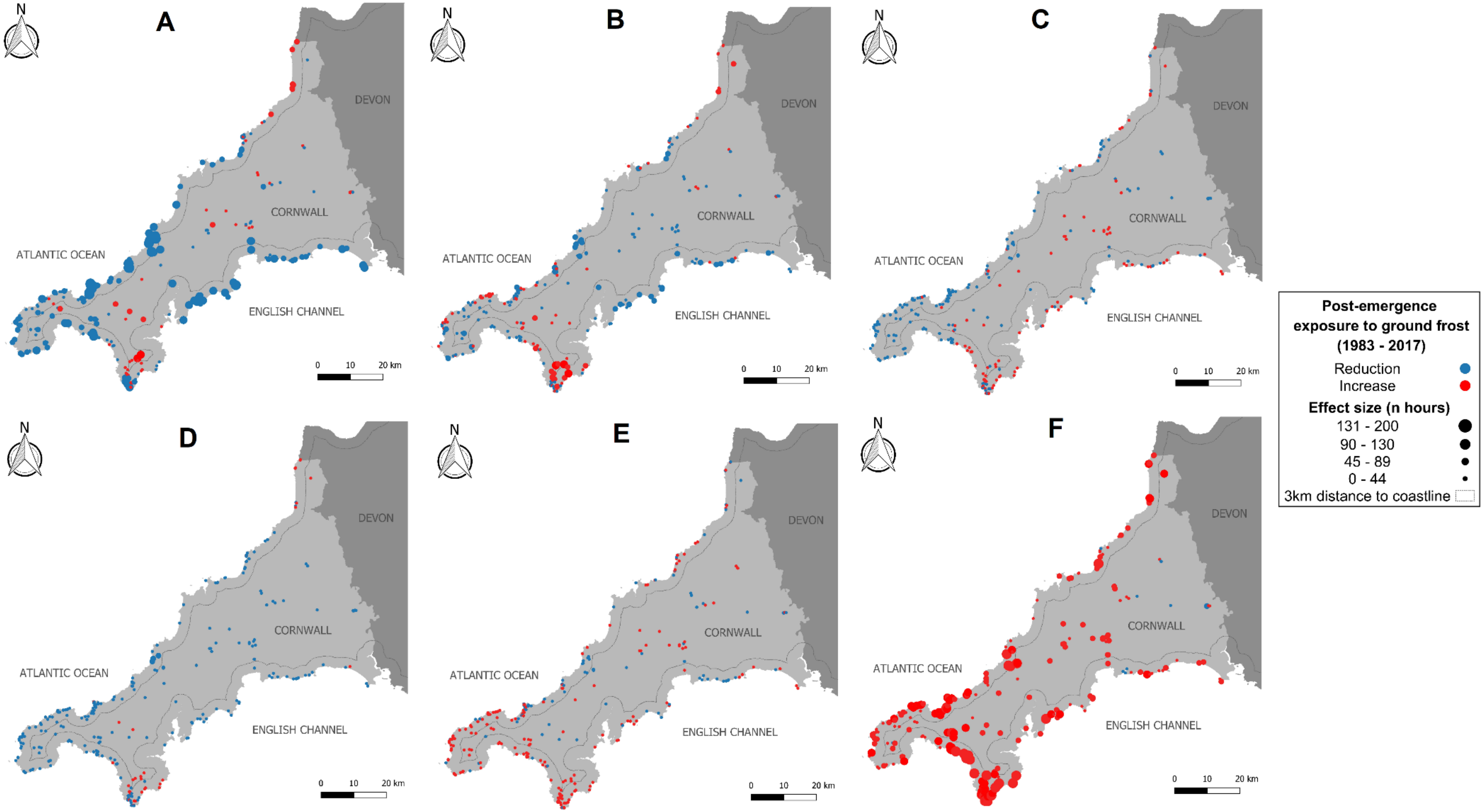

Figure S5. Trends in annual post-emergence ground frost exposure for *Vipera berus* at sites in Cornwall, United Kingdom, with known historical occupancy (1983–2017) (n = 344) using alternative cues and thresholds for *V. berus* spring-emergence: (A) 2.5th percentile value of accumulated degree-hours; (B) 5th percentile value of accumulated degree-hours as presented in the main text; (C) 10th percentile value

of accumulated degree-hours; (D) Sharpest rise in accumulated degree-hours; (E) Below-ground temperature gradient collapse; (F) 10°C Critical air temperature.

The mechanism underpinning a climate trap appeared similar to that presented in the main text under all alternative values of accumulated degree-hours thresholds to predict emergence. Whether sites experience a trend towards greater or lesser exposure to ground frost post-emergence appeared to be governed by the rate at which spring ground frost depletes over the course of the year, relative to the rate at which thermal energy (degree-hours) accumulates. This is demonstrated in Fig. S6, whereby at exemplar inland and coastal sites, with opposing trends in post-emergence exposure to ground frost, the mechanism leading to a heightened risk of a climate trap using the 2.5th degree-hours threshold (Fig S6ii) is comparable to that of the 5th percentile (Fig S6i). The impact of warming was more evident when using the 2.5th percentile cue but less discernible when using the 10th percentile threshold (Fig S6iii). The sharpest rise in accumulated degree-hours cue depicted a climate trap mechanism (Fig S7) comparative to that derived using the 2.5th and 5th percentile values of accumulated degree-hours.

There were contrasting results in the number of potential climate traps observed when emergence was assumed to be determined by either the hibernacula gradient collapse cue or the 10°C critical air temperature cue. Under the assumption that emergence follows a collapse in the hibernacula temperature gradient, this risk appears to be determined by the effects of warming on the stability and strength of below-ground temperatures (Fig. S8). For instance, as shown in Fig. S8, increased exposure to ground frost is actually more likely to occur under gradual warming producing milder temperatures, rather than when warming occurs rapidly and produces higher ambient temperatures. Under gradual warming, spring temperatures are milder and therefore the risk of encountering ground frost may be more ambiguous to adders, resulting in earlier emergence and a pronounced increase in exposure to ground frost (Fig. S8). Likewise, milder spring air temperatures may also lead to ambiguity, as post-emergence exposure to ground frost was higher under gradual warming when a 10°C critical air temperature was assumed to cue emergence (Fig. S9). Under a strong effect of warming, the amount of ground frost occurring in the spring is significantly reduced and emergence is predicted to occur later in the year (Figs. S8-9). This can perhaps be explained by the slower rate of temperature gradient collapse and that temperature rises at a slower rate in the spring in a warm year. For instance, in a spring preceded by a warm winter, the difference in above- and below-ground temperatures is less pronounced, the range of colder air temperatures is reduced and the hibernacula temperatures remain stable for longer before more rapid warming occurs later in the spring. On the other hand, in a spring with mild temperatures, the temperature gradient drops off at a faster rate and air temperatures increase more rapidly under warming, particularly if preceded by a cold winter. This results in earlier emergence, at a time in the season when the likelihood of ground frost is higher (Figs S8-9).

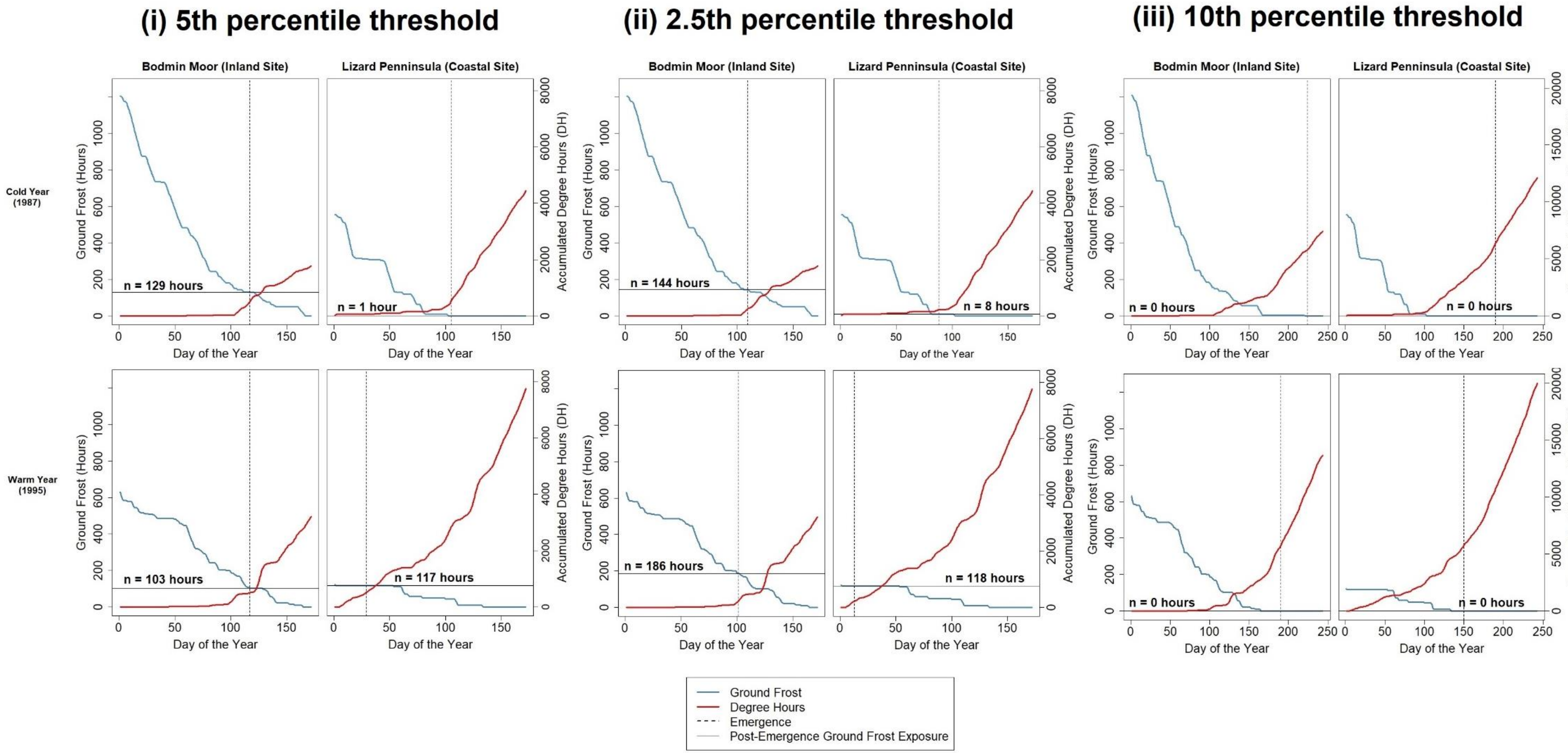

Figure S6. Estimated *Vipera berus* emergence timing, temperature accumulation (degree-hours) and ground frost depletion in warm and cold years at two *V. berus* sites in Cornwall, United Kingdom, with known historical occupancy. The results for one site situated on Bodmin Moor (an inland site with reduced post-emergence ground frost between 1983 and 2017) and one site situated on the Lizard Penninsula (a coastal site with increased post-emergence ground frost between 1983 and 2017) are presented. Alternative thresholds of accumulated degree-hours triggering *V. berus* spring-emergence were used: (i) 5th percentile value of accumulated degree-hours; (ii) 2.5th percentile value of accumulated degree-hours; (iii) 10th percentile value of accumulated degree-hour.

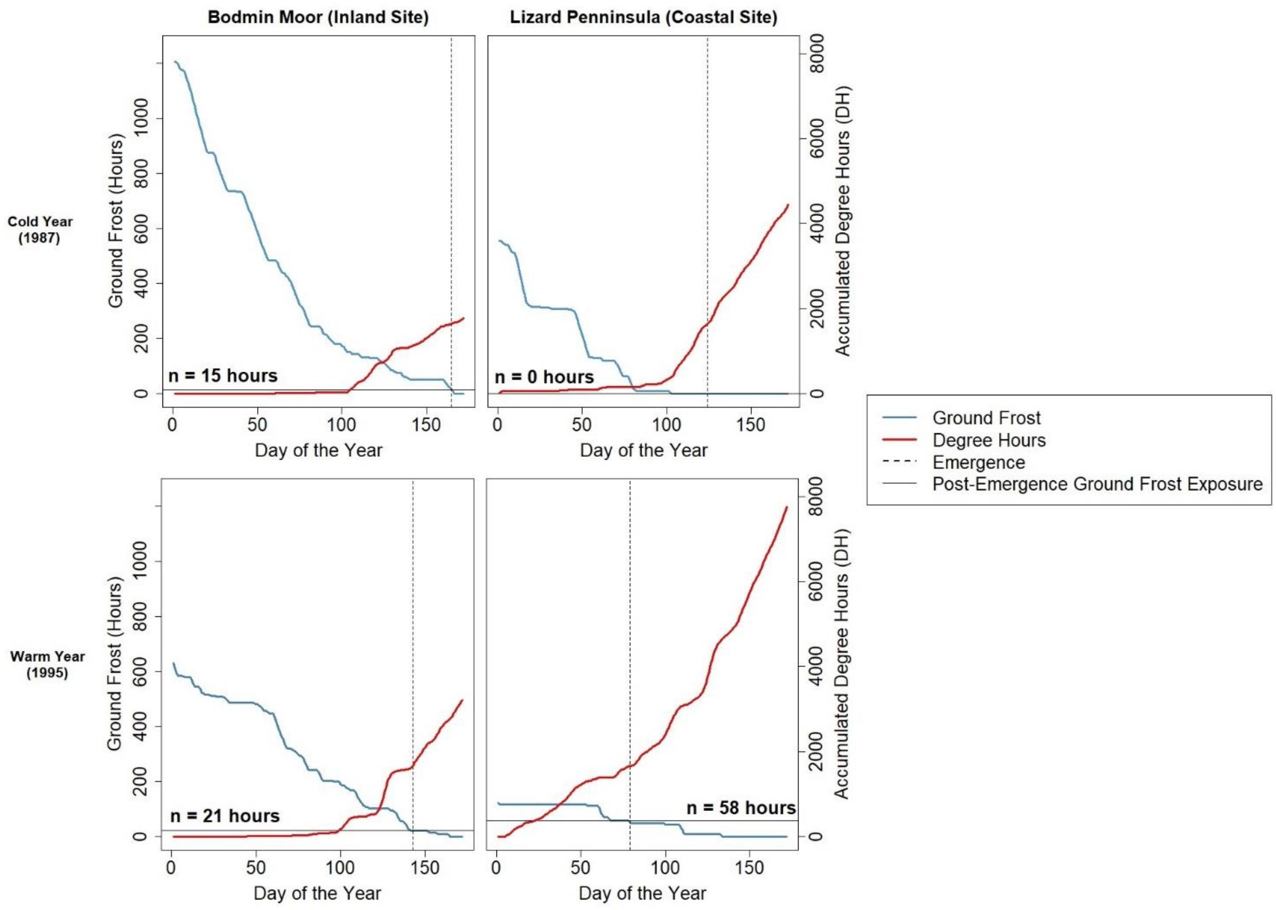

Figure S7. Estimated *Vipera berus* emergence timing using the sharpest rise in accumulated degree-hours cue, temperature accumulation (degree-hours) and ground frost depletion in warm and cold years at two *V. berus* sites in Cornwall, United Kingdom, with known historical occupancy. The results for one site situated on Bodmin Moor (an inland site with reduced post-emergence ground frost between 1983 and 2017) and one site situated on the Lizard Penninsula (a coastal site with increased post-emergence ground frost between 1983 and 2017) are presented.

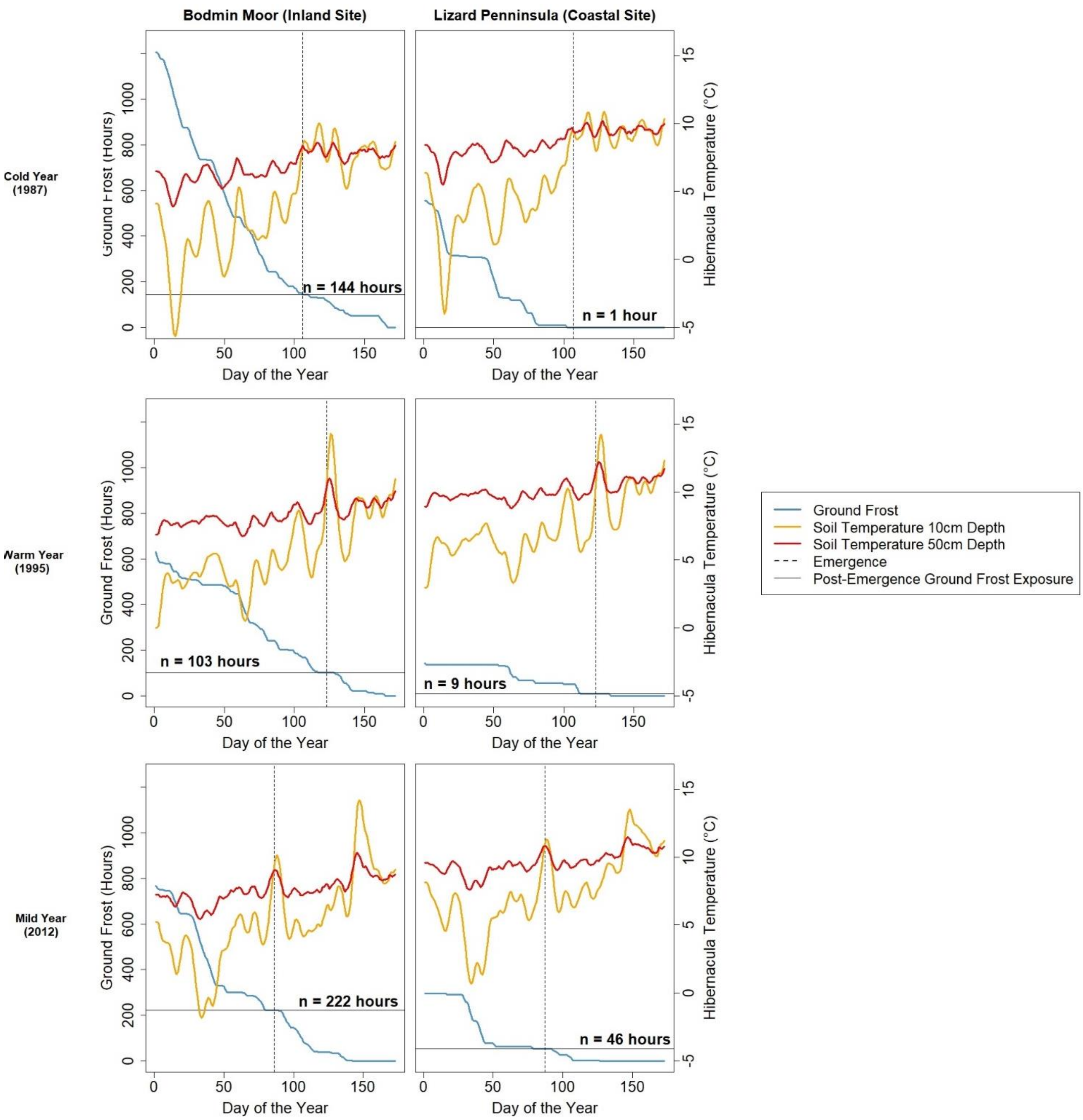

Figure S8. Estimated *Vipera berus* emergence timing using a below-ground temperature gradient collapse as the cue for *V. berus* spring-emergence, daily 2.5-day moving average of hibernacula temperature gradients (temperatures at 10 cm and 50cm depths), and ground frost depletion in cold, warm and mild years at two *V. berus* sites in Cornwall, United Kingdom, with known historical occupancy. The results for one site situated on Bodmin Moor (an inland site with reduced post-emergence ground frost between 1983 and 2017) and one site situated on the Lizard Penninsula (a coastal site with increased post-emergence ground frost between 1983 and 2017) are presented.

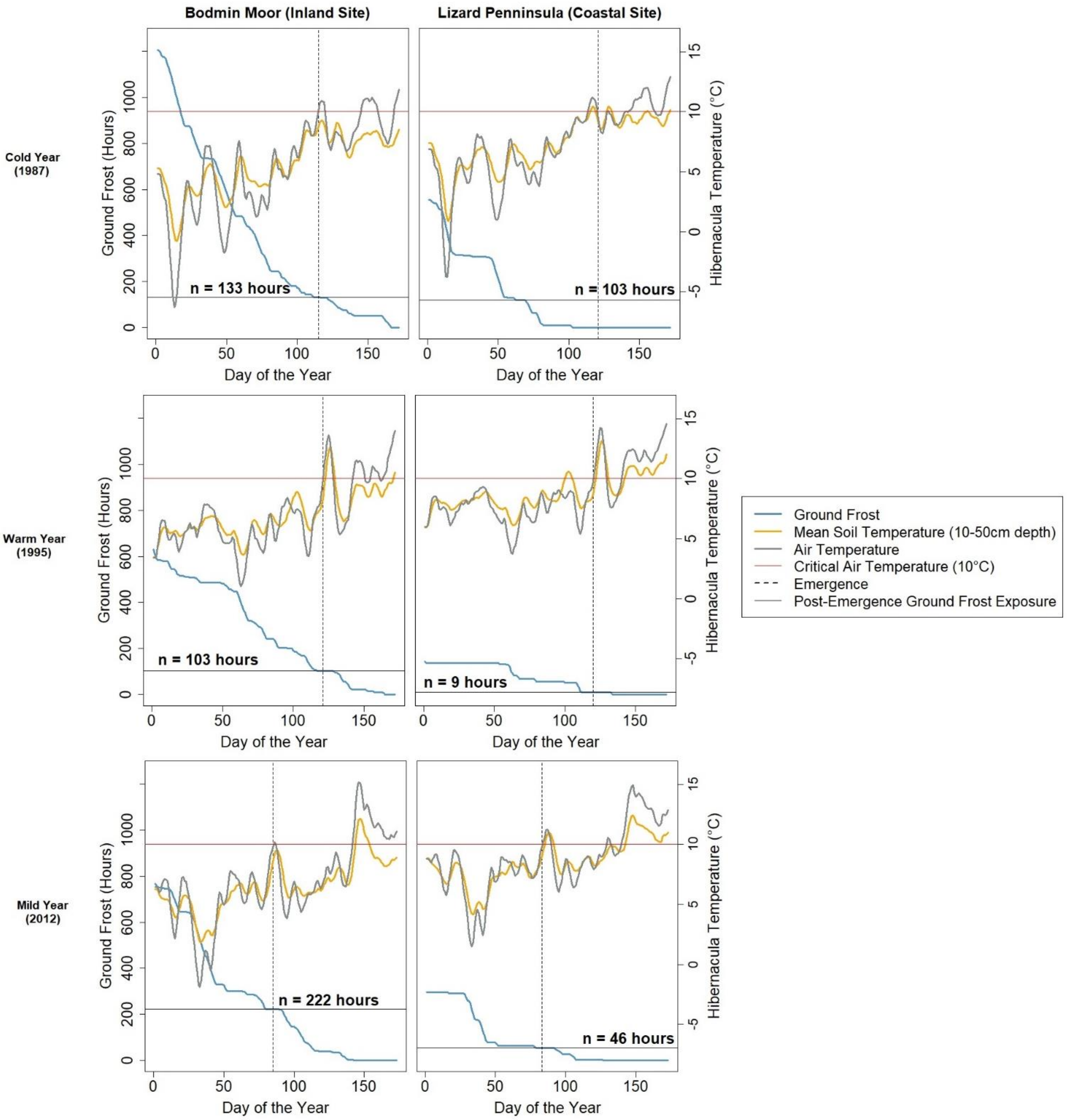

Figure S9. Estimated *Vipera berus* emergence timing using a 10°C critical air temperature as the cue for *V. berus* spring-emergence, the daily 2.5-day moving average of air temperatures, the daily 2.5-day moving average of hibernacula temperatures (the mean of soil temperatures at 10cm and 50cm depths) and ground frost depletion in cold, warm and mild years at two *V. berus* sites in Cornwall, United Kingdom, with known historical occupancy. The results for one site situated on Bodmin Moor (an inland site with reduced post-emergence ground frost between 1983 and 2017) and one site situated on the Lizard Penninsula (a coastal site with increased post-emergence ground frost between 1983 and 2017) are presented.

Additional references not cited in the main manuscript

Brito, J. C. (2003). Seasonal and daily activity patterns of Vipera latastei in northern Portugal. *Amphibia-Reptilia*, *24(4)*, 497–508.

Litzgus, J. D., Costanzo, J. P., Brooks, R. J., & Lee Jr., R. E. (1999). Phenology and ecology of hibernation in spotted turtles (Clemmys guttata) near the northern limit of their range. *Canadian Journal of Zoology*, *77*, 1348–1357.
